# Can We Harness “Enviromics” to Accelerate Crop Improvement by Integrating Breeding and Agronomy?

**DOI:** 10.3389/fpls.2021.735143

**Published:** 2021-09-10

**Authors:** Mark Cooper, Carlos D. Messina

**Affiliations:** ^1^Queensland Alliance for Agriculture and Food Innovation (QAAFI), The University of Queensland, Brisbane, QLD, Australia; ^2^Corteva Agriscience, Johnston, IA, United States

**Keywords:** environmental characterisation, envirotyping, yield prediction, drought, crop modelling, crossover genotype by environment interactions, target population of environments, multi-environment trial

## Abstract

The diverse consequences of genotype-by-environment (GxE) interactions determine trait phenotypes across levels of biological organization for crops, challenging our ambition to predict trait phenotypes from genomic information alone. GxE interactions have many implications for optimizing both genetic gain through plant breeding and crop productivity through on-farm agronomic management. Advances in genomics technologies have provided many suitable predictors for the genotype dimension of GxE interactions. Emerging advances in high-throughput proximal and remote sensor technologies have stimulated the development of “enviromics” as a community of practice, which has the potential to provide suitable predictors for the environment dimension of GxE interactions. Recently, several bespoke examples have emerged demonstrating the nascent potential for enhancing the prediction of yield and other complex trait phenotypes of crop plants through including effects of GxE interactions within prediction models. These encouraging results motivate the development of new prediction methods to accelerate crop improvement. If we can automate methods to identify and harness suitable sets of coordinated genotypic and environmental predictors, this will open new opportunities to upscale and operationalize prediction of the consequences of GxE interactions. This would provide a foundation for accelerating crop improvement through integrating the contributions of both breeding and agronomy. Here we draw on our experience from improvement of maize productivity for the range of water-driven environments across the US corn-belt. We provide perspectives from the maize case study to prioritize promising opportunities to further develop and automate “enviromics” methodologies to accelerate crop improvement through integrated breeding and agronomic approaches for a wider range of crops and environmental targets.

## Introduction

Sustainable improvement of on-farm crop yield productivity, through improving yield potential and yield stability, is a complex long-term objective for both breeders and agronomists (Duvick et al., 2004; Hall and Richards, 2013; Fischer et al., 2014; Hatfield and Walthall, 2015; Beres et al., 2020; Cooper et al., 2021; Hunt et al., 2021). Heterogeneity of current environmental conditions that impact crop yield and the influences of climate change continually challenge the definition of the Target Population of Environments (TPE) for both breeders and agronomists (Chapman et al., 2012; Harrison et al., 2014; Lobell et al., 2015; Voss-Fels et al., 2019; Hammer et al., 2020; Cooper et al., 2021; Smith et al., 2021). Many of the important environmental details required for interpretation of experimental results and to enable prediction of genotype reaction-norms are not currently captured routinely for the multi-environment trials (METs) conducted by breeders and agronomists. Further, for most crop breeding programs the relationships between the environments sampled in METs and the dominant environmental conditions of the TPE are neither well understood nor adequately quantified (Cooper and DeLacy, 1994; Cooper et al., 2021). Improved sensor technologies and prediction methodologies are urgently required to characterize and study environments within breeding and agronomy METs and to quantify the relationships between the environments sampled in METs for all stages of crop improvement programs and their importance for the TPE (Messina et al., 2020; Crespo-Herrera et al., 2021; Kusmec et al., 2021; Potgieter et al., 2021; Smith et al., 2021).

Genotype-by-environment interactions (GxE) have been long recognized as important factors impacting successful application of selection in plant breeding and for the yield stability of cultivars released from breeding programs (Comstock and Moll, 1963; Finlay and Wilkinson, 1963; Allard and Bradshaw, 1964; Eberhart and Russell, 1966; Blum, 1988; Nyquist and Baker, 1991; Cooper and DeLacy, 1994; Cooper et al., 2020). Similarly, agronomists have a long history of investigating the environmental responses of cultivars developed by breeding programs under on-farm management systems (French and Schultz, 1984; Passioura, 2002, 2006, 2007; Sadras and Angus, 2006; Kirkegaard and Hunt, 2010; Van Ittersum et al., 2013; Holzworth et al., 2014; Assefa et al., 2018; Archontoulis et al., 2020; Hunt et al., 2021). Farmers seek improved technology combinations based on genotypes and agronomic management that can consistently deliver yield productivity close to the potential of their on-farm environments, while managing the risk of crop failure (Hammer et al., 2014; Hunt et al., 2021). As a constructive step toward improving the predictability of on-farm crop productivity, there has been continual refinement of the definition of environments in METs and the agricultural TPE to recognize the important role of crop management and for investigation of the influences of genotype-by-environment-by-management (GxExM) interactions (Kirkegaard and Hunt, 2010; Hammer et al., 2014, 2020; Hatfield and Walthall, 2015; Beres et al., 2020; Peng et al., 2020; Cooper et al., 2021; Hunt et al., 2021; Potgieter et al., 2021; Smith et al., 2021). Thus, we can study genetic improvements from the perspective of the breeder, crop management improvements from the perspective of the agronomist, and improvement in genotype–management technology combinations from the perspective of the farmer. In all cases, an improved understanding of the environmental context for achievable yield performance can enhance their contributions to further improve on-farm crop productivity. Hence the importance of the nascent technologies and methods of enviromics.

## Perspective: Harnessing Enviromics for Crop Improvement

While the use of the terminology “enviromics” is relatively recent, the motivations and concepts for studying agricultural environments in METs and the TPE to accelerate crop improvement have a long history. There have been many calls for enhanced attention to environmental characterization to accelerate crop improvement. Plant breeders have long sought environmental definitions and covariates to assist interpretation of plant responses and the GxE interactions detected in METs and to understand their relevance for the on-farm TPE (Finlay and Wilkinson, 1963; Allard and Bradshaw, 1964; Baker, 1988; Blum, 1988; Cooper and Hammer, 1996; Boer et al., 2007; Heslot et al., 2014; Jarquín et al., 2014; Pauli et al., 2016; Xu, 2016; Ly et al., 2018; Bustos-Korts et al., 2019, 2021; Millet et al., 2019; Costa-Neto et al., 2020, 2021; Porker et al., 2020; Crossa et al., 2021; Li et al., 2021; Resende et al., 2021; Smith et al., 2021). The role of water availability and impact of drought on crop yield and investigations to determine the traits contributing to crop productivity under drought conditions have received significant attention from breeders (e.g., Blum, 1988; Fukai and Cooper, 1995; Campos et al., 2004; Bänziger et al., 2006; Ribaut, 2006; Messina et al., 2011, 2018; Cooper et al., 2014a), agronomists (French and Schultz, 1984; Sadras and Angus, 2006; Kirkegaard and Hunt, 2010; Van Ittersum et al., 2013; Hunt et al., 2021), and physiologists (Richards and Passioura, 1989; Ludlow and Muchow, 1990; Bolaños and Edmeades, 1996; Passioura, 2002, 2006, 2007; Messina et al., 2011, 2015, 2019; Araus and Cairns, 2014; Hammer et al., 2014; Araus et al., 2018; Sinclair, 2018; Simmons et al., 2021). Complexity, cost, and the timeliness of detailed measurements of the water status of environments and genotypic variation for plant responses to water deficits under field conditions have limited adoption and application of many discoveries and methods to the scale of breeding programs. Recently, new proximal and remote sensor technologies and data modelling capabilities have become available to enhance characterization of environments and measure plant responses under field conditions at higher throughput and at greater scales to enhance applications for crop improvement and yield prediction (Pauli et al., 2016; Guan et al., 2017; Araus et al., 2018; Messina et al., 2018; Van Eeuwijk et al., 2019; Cooper et al., 2020; Messina et al., 2020; Peng et al., 2020; Schwalbert et al., 2020; Costa-Neto et al., 2021; Jain et al., 2021; Jin et al., 2021; Potgieter et al., 2021; Smith et al., 2021; Yang et al., 2021).

Terminology has emerged in combination with the advances in the technologies for studying the characteristics of environments in METs, for their applications to assist interpretation of GxE interactions, and to quantify reaction-norms for genotypes. To ensure we benefit from the deep history of studying agricultural environments and how they influence plant responses, crop performance, and adaptation for the TPE, we include what has previously been referred to as environmental characterization (Fukai and Cooper, 1995; Chapman et al., 2000; Löffler et al., 2005; Chenu et al., 2011; Mathews et al., 2011; Kholová et al., 2013; Pauli et al., 2016; Smith et al., 2021) and envirotyping (Cooper et al., 2014b; Pauli et al., 2016; Xu, 2016; Porker et al., 2020; Couëdel et al., 2021; Smith et al., 2021) within scope of the applications of enviromics for crop improvement. The convention we adopt is that the terminology of *enviromics* represents the collective of activities that are undertaken to study, measure, and quantify the characteristics of micro- and macroenvironments and how they influence responses of plants (genotypes) at the field, MET and TPE levels. Within the domain of enviromics, the terminology of *environmental characterization* is used to refer to the applied activities that use the methodologies and technologies of enviromics to characterize the important environmental variables that are influential on the plant responses observed within field conditions and experimental METs. Further, the concept and terminology of *envirotyping* are applied to uses of the available environmental characterization information to identify appropriate groupings of the environments sampled in METs and to quantify their relationships to the TPE to assist interpretations of plant responses to the environments, any GxE interactions, and differences in reaction-norms of genotypes at the levels of the MET and the TPE. The level of envirotyping resolution that can be applied extends along a continuum from coarse-grained to fine-grained, depending on the target situation (Cooper et al., 2014a,b, 2020). We encourage the constructive transdisciplinary dialogue that is required to provide an improved understanding of the environmental variables that determine important GxE interactions and ultimately identification of sets of coordinated environmental and genomic predictors of variation for genotypic reaction-norms within the MET and the TPE (e.g., Messina et al., 2011; Ly et al., 2018; Bustos-Korts et al., 2019, 2021; Millet et al., 2019).

## Example: Harnessing Enviromics for Maize Yield Improvement in the US Corn-Belt

The genetic improvement in grain yield of temperate maize for the US corn-belt provides a useful case study for considering past and potential roles of enviromic technologies to contribute to strategies focused on accelerating yield improvement for the future TPE. Past contributions to improvements in on-farm yield productivity of maize from both genetics and agronomy have been documented (Russell, 1991; Duvick et al., 2004; Duvick, 2005; Cooper et al., 2014a). The influences of GxE and GxExM interactions on grain yield variation have been investigated (Boer et al., 2007; Messina et al., 2009; Gaffney et al., 2015; Assefa et al., 2018; Cooper et al., 2020; Rogers et al., 2021). Environmental heterogeneity within the TPE and its influence on GxE interactions for yield have been quantified (Löffler et al., 2005; Cooper et al., 2020; Crespo-Herrera et al., 2021; Rogers et al., 2021) and the important influence of drought on grain yield recognized (Boyer et al., 2013; Gaffney et al., 2015; Kimm et al., 2020). The environmental and genetic determinants of GxE interactions for grain yield of maize have been investigated by variance components, stability analysis, and more recently through extensions of these approaches using molecular markers and crop models (Eberhart and Russell, 1966; Boer et al., 2007; Gage et al., 2017; Messina et al., 2018; Cooper et al., 2020; Rogers et al., 2021). Agronomic management strategies that reduce on-farm yield gaps have been developed (Grassini et al., 2011; Assefa et al., 2018). There is ongoing interest in using improved understanding of the environmental determinants of yield performance, adaptation, and reaction-norms of maize hybrids that provide a focus for testing and further development of enviromic methodologies (Cooper et al., 2014a,b, 2020; Gage et al., 2017; Messina et al., 2020; Kusmec et al., 2021; Rogers et al., 2021).

**Figure 1:**
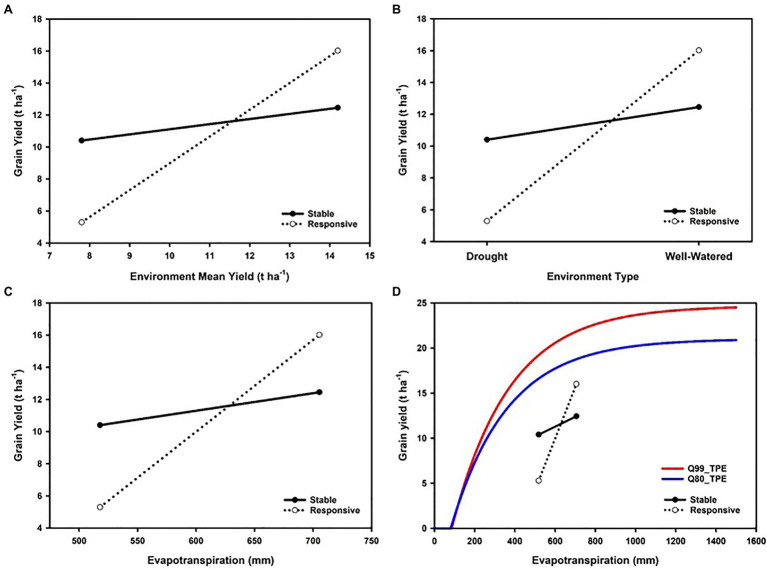
Enviromics progression from **(A)** coarse-grained to **(D)** fine-grained characterization of environmental gradients for a maize multi-environment trial (MET) to assist interpretation of grain yield genotype-by-environment (GxE) interactions and genotypic variation for reaction-norms: **(A)** environments distinguished on mean grain yield of all genotypes tested (e.g., Finlay and Wilkinson, 1963), **(B)** environments distinguished on levels of water inputs; water-limited (drought) versus water-sufficient (well-watered), **(C)** environments distinguished on levels of water availability quantified as whole season crop evapotranspiration, **(D)** environments distinguished on evapotranspiration and considered in relation to the modelled 99th percentile and 80th percentile yield-evapotranspiration fronts for modelled genotype-by-environment-by-management (GxExM) scenarios for the US corn-belt following the methodology of Cooper et al. (2020).

Trait GxE interactions identified from the results of METs can be investigated in terms of models of the reaction-norms of genotypes across an environmental gradient (Figure 1A). When modelling genotype reaction-norms, in the absence of informative descriptors to order the environments, the mean yield of all genotypes that were tested in an environment has been used as an environmental gradient for such investigations, e.g., Finlay and Wilkinson (1963), Allard and Bradshaw (1964), and Eberhart and Russell (1966) are early examples. In such cases, there has always been a recognition of the need for more informative environmental descriptors to enhance the predictive skill of models for new environments outside of the sample obtained in METs.

An important distinction is drawn between GxE interactions that are a consequence of differences in magnitude of genetic variance among environments and those that result in changes in the rank of the genotypes across the environmental gradient (Baker, 1988; Cooper and DeLacy, 1994; Van Eeuwijk et al., 2001, 2016). Such analyses can be applied to the empirical results and genomic predictions for any stage of a breeding program (Cooper et al., 2014a,b). When water availability is recognized as an important contributor to the differences in mean yield levels of environments, the environmental gradient can be investigated and characterized in terms of environmental descriptors of water availability, e.g., plant available water content in the soil, and crop evapotranspiration. Many approaches have been attempted, ranging from coarse-grained to fine-grained characterization of environmental differences in water availability. A common coarse-grained approach is to categorize environments as either water-limited (drought) or water-sufficient (irrigated or well-watered), e.g., Gaffney et al. (2015) (Figure 1B). To complement such environment characterization of METs, plant breeders and agronomists have conducted controlled side-by-side experiments imposing treatments based on levels of water inputs through managing irrigation levels to represent water-deficit and water-sufficient environments expected in the TPE. When drought is of sufficient importance in the TPE to become a long-term breeding target, this has in some cases justified the establishment of specialized field-based research facilities to enable more fine-grained consideration of the continuum of environments ranging from water-deficient to water-sufficient (e.g., Fischer et al., 1989; Cooper et al., 1995, 2014a, 2020; Weber et al., 2012; Rebetzke et al., 2013).

Enviromic technologies have been incorporated within the operations of such dedicated field-based drought research facilities to enable the detailed characterization of the environmental conditions within experiments (Cooper et al., 2014a; Reynolds et al., 2020) and to understand and predict important GxExM interactions at the different stages of a breeding program (Cooper et al., 2014a,b). Such integration of enviromic technologies into breeding operations has enabled definition and quantification of key environmental variables, detailed studies of trait contributions to yield variation within breeding program cycles and prediction of trait contributions to yield improvement for the TPE (Messina et al., 2011, 2015, 2018; Cooper et al., 2014a). The upscaling of the environmental characterization of water availability in drought experiments, based on proximal and remote sensor technologies, has been enabled through using the environmental measurements directly, e.g., vapor pressure deficit, evapotranspiration, rainfall, temperature, or as inputs to crop models to quantify daily water balance throughout the crop life cycle, from planting to harvest (French and Schultz, 1984; Chapman et al., 2000; Sadras and Angus, 2006; Gaffney et al., 2015; Messina et al., 2015). The integrated use of the environmental measurements with a suitable crop model (e.g., Messina et al., 2019) enables a continuum of coarse-grained to fine-grained characterization of environments. Recent applications of the integrated sensor and crop modelling approach have investigated characterization of environmental water sufficiency in terms of crop level evapotranspiration and the timing of water deficits in relation to crop growth and development using the concept of crop-level water supply/demand ratio determined on a daily time step (Muchow et al., 1996; Chapman et al., 2000; Chenu et al., 2011; Messina et al., 2015; Cooper et al., 2020). Therefore, using such advances in enviromic capabilities to characterize breeding and agronomy METS, the environmental gradient used to study genotypic reaction-norms can be refined from the coarse-grained view of a contrast between water-limited and water-sufficient (Figure 1B) and quantified in terms of important environmental variables, such as the crop-level evapotranspiration (Figure 1C).

Environmental descriptors such as seasonal crop-level evapotranspiration also have been extensively used by agronomists to study the expected yield potential of crops based on water availability for the range of environments that comprise a TPE and to quantify the yield-gaps between the yield potential and the on-farm water-limited yield levels that are achieved by farmers (French and Schultz, 1984; Sadras and Angus, 2006; Van Ittersum et al., 2013; Fischer et al., 2014; Sadras et al., 2015). A curated global yield-gap atlas is available for a range of crops (Van Bussel et al., 2015).^1^ Applying the methodology for yield-gap analysis, Cooper et al. (2020) developed a water-limited yield front for the US corn-belt by parameterizing a crop model for a range of maize hybrids. The water-limited yield fronts they obtained represent a yield potential reaction-norm where yield was related to in-season crop evapotranspiration.

**Figure 2:**
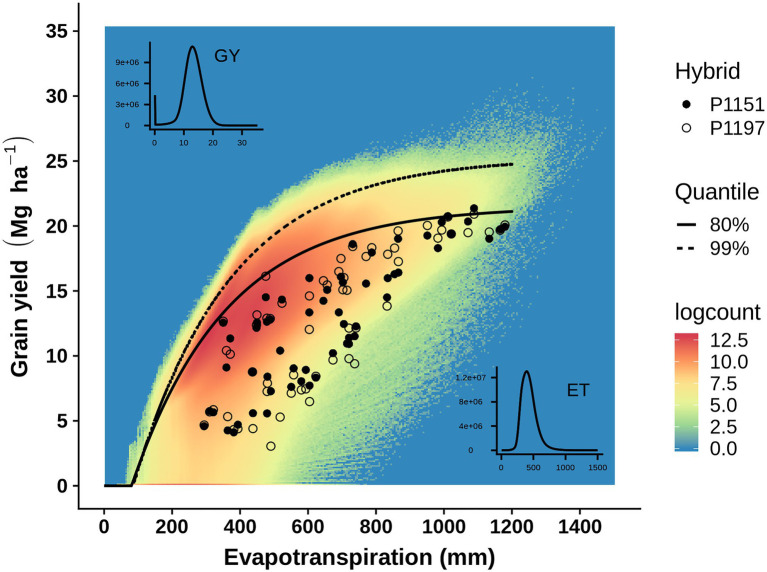
Enviromics applied to assess how well a MET represents a target population of environments (TPE). Example of empirical grain yield results from a maize MET compared to GxExM expectations for the US corn-belt TPE. To compare the empirical MET results and modelled TPE expectations, environments were characterized in terms of crop evapotranspiration to quantify the gradient from water-limited (low evapotranspiration) to water-sufficient environments (high evapotranspiration). Enviromics approaches were applied to the environments sampled in the MET to obtain the inputs for a crop model, which was used to estimate crop evapotranspiration following Cooper et al. (2020). The MET example focuses on the yield comparison between two hybrids, P1197 (Responsive; Figure 1) and P1151 (Stable; Figure 1). The empirical yield-evapotranspiration results for the MET are superimposed on the simulated cloud of yield-evapotranspiration outcomes for the TPE. The estimates of the 99th and 80th percentile yield-evapotranspiration fronts for the TPE provide a reference for interpreting the empirical yield-evapotranspiration results hybrid reaction-norms obtained for the MET. Whenever the empirical yield for a GxExM combination falls below the 80th percentile yield-evapotranspiration front a yield-gap is associated with the on-farm yield. The empirical results from the MET can then be analyzed to identify on-farm situations where the yield-gap can be reduced by choice of genotype (e.g., stable or responsive), agronomic management (e.g., plant density, irrigation strategy), or genotype–management technology combinations.

Crop evapotranspiration provides a useful environmental descriptor to study GxE interactions in plant breeding METs and to study GxExM interactions and yield-gaps in agronomy METs. Therefore, given suitable enviromic technologies to measure crop evapotranspiration (Guan et al., 2017; He et al., 2019; Cooper et al., 2020) a common view of genotypic reaction-norms for breeding and agronomic applications can be constructed (Figure 1D). Applying yield-evapotranspiration fronts estimated for maize in the US corn-belt, Cooper et al. (2020) investigated the opportunities to close yield-gaps from an integrated breeding and agronomic perspective. With availability of genotypic and environmental predictors, such an integrated view of GxExM interactions can be predicted for all stages of a plant breeding program to inform selection and hybrid advancement by breeders and to assist agronomists to provide decision support services to identify suitable combined genotype and management technologies for farmers to reduce on-farm yield productivity gaps (Cooper et al., 2014b). Figure 2 provides an example of such an integrated view, constructed by superimposing a yield-evapotranspiration front modelled for the TPE of the US corn-belt and the empirical yield results for two contrasting maize hybrids that were obtained from a MET where an enviromic approach was applied to quantify the range of crop evapotranspiration levels sampled in the MET. With this integrated view the GxExM interactions associated with the empirical yield results from the MET can be investigated from a breeding perspective selecting for improved yield potential and yield stability and from an agronomy perspective to identify genotype and management technology combinations to close yield-gaps given the crop available water and the achievable yield for an environment.

Improvements in proximal and remote sensor technologies to quantify and upscale measurement of important environmental variables determining GxExM interactions, e.g., evapotranspiration (Figure 2; Guan et al., 2017; He et al., 2019; Kimm et al., 2020), open a wide range of opportunities for applications of enviromic technologies to accelerate crop improvement by integrating breeding and agronomy (Cooper et al., 2020; Peng et al., 2020; Kusmec et al., 2021) and enabling environment-specific predictions (Rogers et al., 2021). For maize breeding in the US corn-belt, early applications of these enviromic technologies have been integrated into the operations of crop improvement programs and are in operational use today (Cooper et al., 2014a,b, 2020; Gaffney et al., 2015; Messina et al., 2018). Such applications of enviromics to analyze GxExM interactions for yield are not restricted to water and drought. Alternative environmental descriptors, such as nitrogen availability (Bänziger et al., 1999; DeBruin et al., 2017; Mueller et al., 2019; Udvardi et al., 2021), can also be applied as appropriate for the crop breeding target, cropping system, and TPE.

## Discussion

Given the ubiquity of GxExM interactions for crop grain yield within an agricultural TPE, it is expected that further developments in the domain of enviromics will continue and their applications will expand as plant breeders incorporate these technologies within their breeding operations. With the continuing advances in crop genomics (Morrell et al., 2012; Yuan et al., 2017; Tao et al., 2021; Varshney et al., 2021) and phenotyping (Araus and Cairns, 2014; Araus et al., 2018; Van Eeuwijk et al., 2019; Messina et al., 2021; Smith et al., 2021), a wide array of suitable genomic predictors are available and becoming cost-effective options for many crop breeding applications. Agronomists and physiologists have invested in the development of methods for measuring important environmental variables (Chenu et al., 2011; Guan et al., 2017; Smith et al., 2021) and suitable crop models to integrate the multiple influences of environmental conditions on yield outcomes for different genotypes (Chapman et al., 2003; Messina et al., 2006, 2018, 2019; Chenu et al., 2009; Holzworth et al., 2014; Muller and Martre, 2019; Wang et al., 2019). These same methods can be developed to provide suitable environmental predictors for envirotyping and to enhance genomic prediction (Cooper et al., 2014a,b; Jarquín et al., 2014; Messina et al., 2018; Voss-Fels et al., 2019; Costa-Neto et al., 2021; Resende et al., 2021). An integrated breeding–agronomy approach to accelerate crop improvement is within reach through operationalizing the genomic, enviromic, phenomics, and quantitative modelling processes required to obtain suitable genotypic and environmental predictors for appropriate stages of crop improvement programs. Successful applications have been demonstrated for commercial maize breeding in the US corn-belt (Cooper et al., 2014a; Gaffney et al., 2015). Opportunities are emerging for development of integrated breeding-agronomy approaches for other crops and target regions to tackle current GxExM challenges and the anticipated impacts of climate change (Hatfield and Walthall, 2015; Hammer et al., 2019; Beres et al., 2020; de los Campos et al., 2020; Messina et al., 2020; Ramirez-Villegas et al., 2020; Crossa et al., 2021; Hunt et al., 2021; Kusmec et al., 2021; Smith et al., 2021; Udvardi et al., 2021).

## Data Availability Statement

The original contributions presented in the study are included in the article/supplementary material, further inquiries can be directed to the corresponding author.

## Author Contributions

MC and CM conceived and wrote the perspective. All authors contributed to the article and approved the submitted version.

## Conflict of Interest

The authors declare that the research was conducted in the absence of any commercial or financial relationships that could be construed as a potential conflict of interest.

## Funding

The contribution was supported by the University of Queensland and the Queensland Alliance for Agriculture and Food Innovation through funding for the Chair of Prediction-based Crop Improvement held by MC.

## Publisher’s Note

All claims expressed in this article are solely those of the authors and do not necessarily represent those of their affiliated organizations, or those of the publisher, the editors and the reviewers. Any product that may be evaluated in this article, or claim that may be made by its manufacturer, is not guaranteed or endorsed by the publisher.

## Footnotes

^1^ https://www.yieldgap.org/
